# Interaction of genetic and environmental factors for body fat mass control: observational study for lifestyle modification and genotyping

**DOI:** 10.1038/s41598-021-92229-5

**Published:** 2021-06-23

**Authors:** Joon Ho Kang, Heewon Kim, Jinki Kim, Jong-Hwa Seo, Soyeon Cha, Hyunjung Oh, Kyunga Kim, Seong-Jin Park, Eunbin Kim, Sunga Kong, Jae-Hak Lee, Joon Seol Bae, Hong-Hee Won, Je-Gun Joung, Yoon Jung Yang, Jinho Kim, Woong-Yang Park

**Affiliations:** 1 Department of Molecular Cell Biology, Sungkyunkwan University School of Medicine, 2066 Seobu-ro, Jangan-gu, Suwon, Gyeonggi-do 16419 South Korea; 2 Samsung Genome Institute, Samsung Medical Center, Sungkyunkwan University, Ilwon-ro 81, Gangnam-gu, Seoul, 06351 South Korea; 3 Department of Foods and Nutrition, College of Natural Sciences, Dongduk Women’s University, 60, Hwarang-ro 13-gil, Seongbuk-gu, Seoul, 02748 Korea; 4 AI&SW Center, SAIT, SEC, 130, Samsung-ro, Yeongtong-gu, Suwon, Gyeonggi 16678 South Korea; 5 Samsung Advanced Institute for Health Sciences and Technology, Sungkyunkwan University of Medicine, Seoul, 06351 South Korea; 6 Samsung Medical Center, Gangnam-gu, Seoul, 06351 South Korea; 7 Department of Clinical Nutrition, School of Public Health, Dongduk Women’s University, Seoul, 02748 Korea; 8 Department of Clinical Research Design and Evaluation, SAIHST, Sungkyunkwan University, Seoul, South Korea

**Keywords:** Computational models, Microarrays, Biomarkers, Health care, Risk factors

## Abstract

Previous studies suggested that genetic, environmental factors and their interactions could affect body fat mass (BFM). However, studies describing these effects were performed at a single time point in a population. In this study, we investigated the interaction between genetic and environmental factors in affecting BFM and implicate the healthcare utilization of lifestyle modifications from a personalized and genomic perspective. We examined how nutritional intake or physical activity changes in the individuals affect BFM concerning the genetic composition. We conducted an observational study including 259 adult participants with single nucleotide polymorphism (SNP) genotyping and longitudinal lifestyle monitoring, including food consumption and physical activities, by following lifestyle modification guidance. The participants’ lifelog data on exercise and diet were collected through a wearable device for 3 months. Moreover, we measured anthropometric and serologic markers to monitor their potential changes through lifestyle modification. We examined the influence of genetic composition on body fat reduction induced by lifestyle changes using genetic risk scores (GRSs) of three phenotypes: GRS-carbohydrate (GRS-C), GRS-fat (GRS-F), and GRS-exercise (GRS-E). Our results showed that lifestyle modifications affected BFM more significantly in the high GRS class compared to the low GRS class, indicating the role of genetic factors affecting the efficiency of the lifestyle modification-induced BFM changes. Interestingly, the influence of exercise modification in the low GRS class with active lifestyle change was lower than that in the high GRS class with inactive lifestyle change (*P* = 0.022), suggesting the implication of genetic factors for efficient body fat control.

## Introduction

Controlling obesity is critical to prevent various types of human diseases, such as type 2 diabetes, cardiovascular diseases, and several cancers^1,2^. However, the prevalence of obesity increased by a large amount—from 3.2 to 10.8% in men, and from 6.4 to 14.9% in women over the past four decades. The general population includes 2.3% and 5.0% of severely obese men and women, respectively^3^. Several genetic studies have demonstrated that genetic factors (genome) are the major association factors for obesity^4–6^. With the rapid advancement of genome analysis techniques, the application of population genomic approaches for determining obesity risk has uncovered several genes and genetic variants in large-scale genome-wide association studies (GWAS). As a result, common variants in the melanocortin 4 receptor (*MC4R*) gene or fat mass- and obesity-associated (*FTO*) genes have been listed as obesity-associated SNPs in the GWAS catalog^7–9^. The association of genes with obesity has been validated in several independent studies^8,10–12^. Several studies have shown that body fat control independently provides health benefits for obesity-related diseases. Visceral adipose tissue or total body fat reportedly displayed a high correlation with obesity-related diseases such as cardiometabolic diseases and type 2 diabetes^13–18^. However, person-specific variations in body fat mass (BFM) with the genetic variations haven’t been examined so far^4^.

Environmental factors, such as dietary habits, smoking, alcohol consumption, and physical activities, reportedly affect weight gain^19,20^, and the impact of diet and/or exercise on body weight control varies^20^. To precisely interpret these associations, genetic factors of obesity should be also analyzed in an integrated manner. Thus, a comprehensive analysis using lifestyle features and genetic information might allow us to understand the fundamental causative factors of weight gain. Previously, we presented a prediction model that can grasp the effect of diet and exercise using the KoGES dataset^21^. We constructed a phenotype (i.e., baseline lifestyle pattern) and genotypic analysis-based genetic risk score (GRS) to understand the efficiency of diet and exercise on BFM. The application of this GRS model allowed us to successfully identify the impact of nutritional factors on BFM according to the significance of genetic factors^22^. Moreover, another study demonstrated that the growth rate in body mass index (BMI) GRS-dependently differed for each nutritional factor^23^.

In order to understand the effect of genetic factors on diet or exercise outcome, systematic analysis, including that of lifestyle changes, would be required. Here, we monitored the BFM of 259 volunteers upon lifestyle modifications in their diet and exercise routine. Our study covered genotyping, anthropometric measurements, serologic marker analysis, and lifelog data, a personal daily record of physical activity, and food-intake, collected from wearable devices throughout the 3-month research period. This study provides a unique and comprehensive view of obesity with lifelog data combined with clinical and genetic-log data.

## Materials and methods

### Experimental design and enrollment

**Figure 1:**
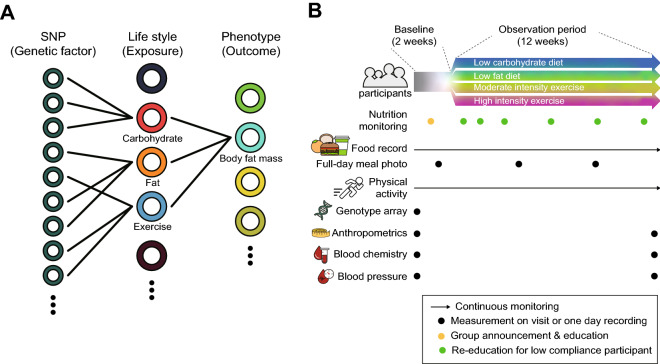
Genome, lifelog, anthropometric characteristics, and blood chemistry profiling. (**A**) Overview of the interaction of genetic and life style factors for body fat mass control. (**B**) Participants were classified into four groups: low-carbohydrate diet, low-fat diet, moderate-intensity exercise and high-intensity exercise groups. Nutrient intake and physical activities were recorded through 14 weeks. Genotypic data were collected at the beginning of the observation. Anthropometrics and blood chemistry data were measured at the beginning and the end of the study.

A total of 259 Korean volunteers (aged 23–67 years, female 22.40%) were enrolled to construct the Samsung Medical Center (SMC) cohort. Participants who desired to improve their health status and control their BFM were recruited in the study. According to their preference, volunteers decided to change their lifestyle, such as exercise or dietary adjustment, for the 3 months of the observation period, following (a) a low-carbohydrate diet (*n* = 35), (b) a low-fat diet (*n* = 34), (c) practicing moderate-intensity exercise (*n* = 99), and (d) practicing high-intensity exercise (*n* = 83) groups (Fig. 1). Each group of the study represents different types of lifestyle modifications to control obesity. Low-carbohydrate or low-fat groups were educated to adjust their nutrient consumption by reducing carbohydrate or fat, respectively. Participants from moderate- and high-intensity exercise groups were recommended to increase the degree or duration of physical activity. Participants with a BMI < 25 were recommended to take part in the exercise modification groups while the rest of the participants with a BMI over 25 were divided into four different groups based on their preference for lifestyle modification. According to lifestyle modification, participants willingly selected, we advised each group to adjust their dietary habits and physical activities, accounting for the baseline. During the baseline period, volunteers were asked to keep their routine diet and report it as a baseline diet pattern. Questionnaires, blood pressure, and anthropometry of individuals on their first visit were recorded to compare with those at the last visit of the study.

### Statement

All experiments and methods were performed in accordance with relevant guidelines and regulations. The experimental procedure was approved by the Institutional Review Board of the Samsung Medical Center (IRB protocol Number: SMC 2017-03-064). Written informed consent was obtained from all participants. The study was conducted from June 2016 to February 2017. This clinical trial was registered at ClinicalTrials.gov (ClinicalTrials.gov ID: NCT04299698) on 05/03/2020.

### Dietary modification

Volunteers recorded their food intake of three meals every day, for 3 months, through the Samsung Health mobile application^24^. The validity of estimating intakes of energy and macronutrients through the Samsung Health mobile application has been reported in detail elsewhere^25^. Photographs of their full daily meals were used to correct and improve the quality of self-recorded food intake data. Nutritionists monitored the food reports to guide the volunteers’ nutrient intake based on the initial data. During the baseline period, volunteers in the diet-adjusting groups were educated on food exchange units, food groups, and healthy macronutrients. Prior to the 3-month observation period, the volunteers were assigned to either the low-carbohydrate or low-fat group. Nutritionists set the target calories based on the volunteers’ height-, weight-, and baseline food consumption and recommended them to change their dietary accordingly. The caloric composition for the low-carbohydrate group was 45:20:35% (carbohydrate: protein: fat%), while that of the low-fat group was 65:20:15%. During the observation period, nutritionists monitored and compared the participant-uploaded meal photographs to the self-reported food intake data and individually counseled the low-compliant participants. All communications were made by telephone.

### Exercise modification

Volunteers were assigned to modify their exercise routine to increase the intensity or duration of their exercises and were asked to wear their wearable devices for constant data collection. Those who were assigned to the moderate-intensity exercise group were asked to exercise at least 150 min per week at an exercise intensity sustained beyond 50% of the heart rate. The members of the high-intensity exercise group were asked to exercise up to 50% of the maximum heart rate for 300 min per week or up to 70% of the maximum heart rate 150 min per week. Volunteers could check their heart rate through their wearable device while exercising, enabling them to control their own exercise intensity.

### Monitoring serologic markers

Thirty-six milliliters of blood were collected from each participant for blood chemistry analysis on their first and last visits. Blood samples were analyzed using COBAS C702 to measure triglycerides, low-density lipoprotein cholesterol (LDLc), and high-density lipoprotein cholesterol (HDLc).

### Collection of lifelog data

To collect the lifelog data of the volunteers, we designed a mobile application, GeneHealth, to record their diet patterns, also monitored by nutritionists to provide feedback. The application provided an interface for recording dietary items in every meal through Samsung Health^24^, based on the FatSecret^26^ and the Korean Nutrition Society^27^ databases. Data on physical activity were collected through the Samsung Galaxy Gear Fit2 Pro upon informed consent.

### Genotyping and quality control

DNA was extracted from blood using the Chemagic™ DNA Blood 200 Kit (PerkinElmer Inc., Waltham, MA, USA). All samples were genotyped for 680,960 SNPs using the Infinium Global Screening Array Kit (Illumina Inc., San Diego, CA, USA). We excluded SNPs for quality control, using the following criteria: call rate < 0.99, minor allele frequency (MAF) < 0.01, and P-value for the Hardy–Weinberg Equilibrium (HWE) < 1.0 × 10^–5^. We also excluded insertion-deletions (indels) and non-autosomal SNPs. After the quality control, a total of 350,522 SNPs were included for imputation. Imputation was conducted using SHAPEIT2 and IMPUTE2, as well as the 1000 Genomes Project phase 3 as the reference.

### Anthropometric measurements

Anthropometric data, including BFM and body fat percentage, were measured using InBody720 (Biospace Co., Ltd.; Seoul, Korea), assessing whole and segmental body compositions before and after the observation^28^. The standard error of body fat mass measured by Inbody 720 calculated by comparison with dual-energy X-ray absorptiometry (DXA) was reported as 0.79–0.99%^29^. All measurements were taken without metal accessories (i.e., with no earring, ring, or belt), shoes, and socks on.

### Questionnaires

Lifestyle information of volunteers was collected using an online or written questionnaire, including data on smoking, frequency and type of alcohol consumption, sweet and salt preferences, fruit and vegetable preferences, oily food preferences, frequency of exercise per week, exercise time, type of exercise, drug history, family disease history, and personal history of the disease.

### Calculation of genetic risk score

For evaluating the genetic predisposition of gaining body fat mass induced by increased intake of macronutrients for each individual, the effect and allele count of genetic variants were integrated into the GRS^30^. GRS-C, GRS-F, and GRS-E represent the genetic susceptibility for estimating the effectiveness of carbohydrate intake, fat intake, and exercise on BFM, respectively. The three GRSs were developed in our previous work^22^. Obesity-associated SNPs were retrieved from the GWAS catalog and tested for interaction between genotype and life-style change on body fat mass change using Korean population data^21^. Generalized linear regression was used for each SNP for evaluating the changes in body fat mass influenced by the interaction between genetic factors and lifestyle changes. The additive genetic effect was assumed for SNP as 0, 1, or 2, which encodes the number of minor alleles. Using gender, age, and the square of age as covariates, we test the effect of the interaction between SNPs and change in carbohydrate intake (ΔC), fat intake (ΔF), or exercise amount (ΔE) on body fat mass.

$$\Delta {\text{Body fat mass }} = {\text{ gender }} + {\text{ age }} + {\text{ age}}^{{\text{2}}} + {\text{ SNP}}_{{\text{i}}} + {\text{ }}\Delta {\text{C }} + {\text{ }}\Delta {\text{F }} + {\text{ }}\beta _{{\text{C}}} \Delta {\text{C }} \times {\text{ SNP}}_{{\text{i}}} + {\text{ }}\beta _{{\text{F}}} \Delta {\text{F }} \times {\text{ SNP}}_{{\text{i}}} ,$$

$$\Delta {\text{Body fat mass }} = {\text{ gender }} + {\text{ age }} + {\text{ age}}^{{\text{2}}} + {\text{ SNP}}_{{\text{i}}} + {\text{ }}\Delta {\text{E }} + {\text{ }}\beta _{{\text{E}}} \times {\text{ SNP}}_{{\text{i}}} .$$

GRS-C, GRS-F, and GRS-E represent the effectiveness of carbohydrate intake changes on body fat mass, the effectiveness of fat intake changes on body fat mass, and the effectiveness of exercise amount changes on body fat mass, respectively. The number of SNPs used for each GRS are 37 for carbohydrate intake; 19 for fat intake; 25 for exercise onset. Each GRS was constructed to count the number of risk alleles of an individual weighted by the coefficient of the interaction between genetic factors and lifestyle changes in the linear model.

### Statistical analysis

To compare the anthropometric measurements, we performed the t-test for continuous variables and the chi-squared test for categorical variables. We performed an ANOVA test to compare the body fat mass changes among the active, inactive lifestyle group and GRS high and low group. P-values < 0.05 were considered statistically significant. All statistics and regression analyses were performed using the R software (version 3.4.2) (http://www.r-project.org/).

### Inclusion and exclusion of the study participants

We recruited healthy or obese volunteers, aged 19–65 years who desired to control their BFM, through the Samsung Medical Center board and online board notices. Among those who took weight control drugs, were diagnosed with diabetes, heart disease, kidney disease, thyroid disease, cancer, or drink excessive amounts of alcohol regularly or quit smoking within a month were excluded from the study. Participants related to the team operating and designing the study at Samsung Electronics Semiconductor Institute within the device solution division in the software development team and Samsung Medical Center were not obliged to enroll in the study.

At the beginning of the enrollment, a total of 399 participants working in various fields were considered to participate in the study. Among them, 379 agreed to have their DNA analyzed. A total of 125 participants who could not show up for the final visit of the research period were excluded from the analysis as the anthropometric and lifelog data were not fully obtained. Also, four participants who are related to each other and one of different ethnicity were excluded. Finally, anthropometric and genotypic data from a total of 259 participants were used for analysis.

## Results

### The evaluation of the lifestyle modification and change of anthropometric and serologic measurement

First, we performed an observational study to test the effectiveness of lifestyle modifications (Fig. 1). Lifestyle modifications were assigned to volunteers according to their preference among the four groups as described in the “Materials and methods” section. Anthropometric measurements and blood chemistry values showed that lifestyle modifications could improve these parameters, as observed in most of the volunteers. BFM, BMI, and LDLc significantly decreased upon diet control and exercise (Table 1). Volunteers showed significant improvement in obesity-related parameters, such as weight, BFM, body fat percentage, BMI, LDLc, and HDLc with P-values < 0.01. Skeletal muscle mass slightly increased, although the increment was not significant. The HDLc levels in the pre- and post-observation periods were beyond 40 mg/dL, which is considered the normal range^31^. Although these values were within the normal range during both periods, they tended to get significantly reduced post-observation.

**Table 1:** Comparison of basic anthropometric and serologic markers of the volunteers between the pre- and post-observation periods.

	Baseline		Post-observation		Differences		P-value
	Mean	s.d.	Mean	s.d.	Mean	s.d.	
**Anthropometric measurement**							
Weight (kg)	73.37	13.06	72.84	13.12	− 0.52	1.94	1.92E−05
Body fat mass (kg)	19.11	6.08	18.54	6.11	− 0.57	1.76	3.57E−07
Body fat percentage (%)	25.89	6.10	25.27	6.11	− 0.62	1.96	7.01E−07
Body mass index (kg/m^2^)	24.81	3.17	24.62	3.18	− 0.19	0.68	7.42E−06
Skeletal muscle mass (kg)	30.42	6.01	30.46	6.01	0.04	0.74	3.54E−01
Waist hip ratio	0.87	0.06	0.87	0.06	0.00	0.02	1.89E−02
**Serologic marker**							
Triglycerides (mg/dL)	128.54	87.55	132.73	100.91	4.18	62.54	2.85E−01
LDL-Cholesterol (mg/dL)	125.21	30.50	120.56	29.72	− 4.65	18.35	6.40E−05
HDL-Cholesterol (mg/dL)	51.93	13.82	50.52	12.83	− 1.41	7.18	1.83E−03

Paired t-test was performed to determine a significant difference between baseline and post-observation period of anthropometric measurements and serologic markers. There were significant differences in weight, body fat mass, body fat percentage, BMI, Waist hip ratio, LDLc, and HDLc.

**Figure 2:**
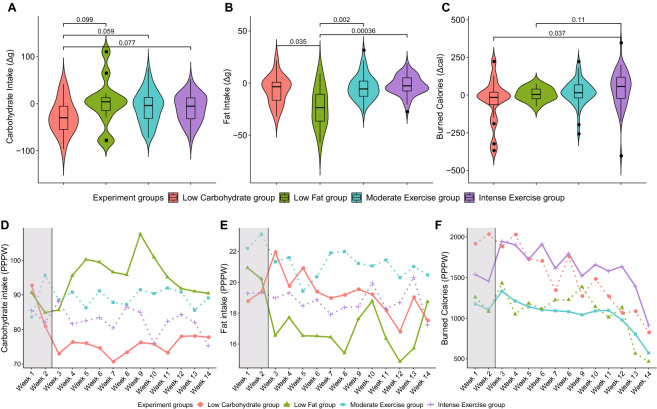
Comparison of caloric expense upon exercise and nutrient intake between the baseline and observation periods. (**A–C**) Differences in the nutrient intake and exercise between the baseline and observation period. (**A**) The differences in the carbohydrate intake (g) between the baseline and observation periods of each group were measured and compared with those of the low carbohydrate group. (**B**) The differences in fat intake (g) between the baseline and observation periods were measured and compared with those of the low-fat group. (**C**) The levels of exercise change between the baseline and observation periods were measured based on their mean caloric expense per day upon exercise. The burned calories per day of the nutrient modification groups were compared with those of the intense-exercise group. (**D–F**) Weekly caloric expense upon exercise and nutrient intake. The area shaded in gray represents the baseline period. (**D**) Mean carbohydrate intake per person per week (PPPW) values through lunch were measured in each group, the carbohydrate intake of the low carbohydrate group being constantly lower during the observation period compared to the other groups. The solid line and dashed line represent weekly caloric expense upon nutrient intake, and exercise, respectively. (**E**) Mean fat intake PPPW values through lunch were measured per each group, and the fat intake was reduced during the observation period in the low-fat group. The solid line and dashed line represent weekly caloric expense upon nutrient intake, and exercise, respectively. (**F**) Mean expanded caloric PPPW values were measured in each group. The intense exercise group shows an overall high caloric expense compared to the other groups. The solid line and dashed line represent weekly caloric expense upon exercise, and nutrient intake, respectively.

We evaluated the lifestyle modifications in each group by calculating the amount of nutrient intake and caloric expense during exercise. The low-carbohydrate and low-fat groups showed further reductions in carbohydrate and fat intake compared to the other groups (Fig. 2A,B). The differences in nutrient intake between the baseline and observation periods for the low-carbohydrate and low-fat groups were as low as − 24.1 and − 16.87 g of carbohydrate and fat, respectively. The participants in both exercise groups increased the intensity of their exercise (Fig. 2C). Intense- and moderate-exercise groups showed a marked increase in caloric expense by 31.21 and 22.75 cal, respectively (Supplementary Table S1, Fig. 2C). We evaluated the compliance of each experimental group by tracking the nutrient intake and exercise amount changes through time (Fig. 2D–F). The mean carbohydrate intake and fat intake per person per week (PPPW) decreased in the low-carbohydrate and low-fat groups during the observation period, compared to the baseline period (Fig. 2D,E). As for the exercise groups, both intense- and moderate-exercise groups increased their caloric expenditure compared to their baseline exercise intensities (Fig. 2F).

### The effect of genetic and environmental factors on BFM changes

We investigated whether the genomic profile of the volunteers affected the effectiveness of lifestyle modification on body fat reduction throughout the observation period. Given that guidance-based lifestyle modification improved the health status of the volunteers, we attempted to analyze the effect of the interaction between genetic and environmental factors. In this analysis, we focused on BFM, which was significantly reduced through the adjustment of nutrient intake and exercise.

**Figure 3:**
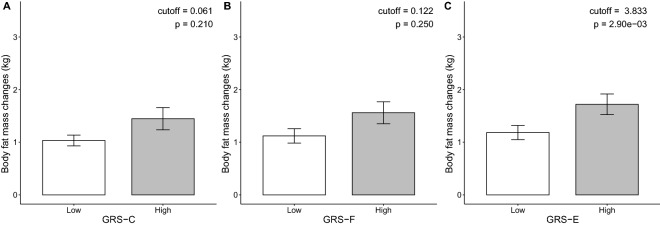
Comparison of the BFM changes between the high and low GRS groups. BFM changes are compared between high and low GRS for each category (GRS-C, GRS-F, and GRS-E). We performed a t-test to compare the mean BFM changes.

To represent the individual genetic susceptibility to BFM changes, we calculated the GRS for each participant using our previously described scoring metho^22^. Here, we calculated three different GRS values by combining allele counts from the individual genotype profiles, weighted by its effect size: (1) GRS-C for estimating the effectiveness of carbohydrate intake on BFM, (2) GRS-F for estimating the effectiveness of fat intake on BFM, and (3) GRS-E for estimating the effectiveness of exercise on BFM. According to the maximal quantitative difference of body fat mass changes, each type of GRS was divided into high- and low-GRS. The cut-off of each GRS classifying high- and low-GRS inferred to the genetic effectiveness of carbohydrate intake, fat intake, and exercise on body fat mass changes the most (carbohydrate = 0.061, fat = 0.122, and exercise = 3.833) (Fig. 3). We then applied statistical tests for each type of GRS to determine the significance of the difference of body fat mass change between high- and low-GRS (T-test; P = 0.210, 0.250, and 2.90E-3, respectively). We performed False Discovery Rate to control type I error on comparison of high and low-GRS-C, F, and E (FDR; 0.250, 0.250, 0.0087, respectively).

**Figure 4:**
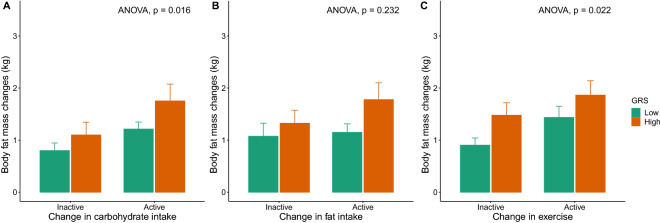
Comparison of the BFM changes between the high and low GRS groups in participants of active and inactive GRS-matched lifestyle change. To better understand GRS efficiency, BFM changes were compared between the active and inactive lifestyle changes. Each extent of the lifestyle changes was compared based on each GRS division.

Having observed a distinct BFM change depending on both GRS classes, we further analyzed BFM efficiency changes according to GRS and GRS-matched lifestyle changes. We classified participants into active and inactive lifestyle change groups, with the pattern of lifestyle modifications. According to participants’ lifelog, the differences of their nutrient intake or exercise intensity between baseline and observation period were quantified and divided by median value to determine participant’s degree of compliance with the given guidelines. In addition, we compared BFM changes in the high- and low-GRS classes for each lifestyle modification. This classification explained the individual BFM change variations (ANOVA; P = 0.016 for carbohydrate intake, 0.232 for fat intake, and 0.022 for exercise) (Fig. 4). These results indicate that the BFM changes increased as the GRS and the amount of GRS-matched lifestyle changes increased. Volunteers with active lifestyle changes and high GRS showed the highest BFM changes compared to those with inactive lifestyle changes and low GRS (Fig. 4). Unexpectedly, BFM changes in high-GRS individuals with inactive lifestyle changes were higher than those of low-GRS individuals with active lifestyle changes in fat intake and exercise amount. This phenomenon was shown to be significant in the case of exercise. This finding suggests that the genetic factors are more efficient in BFM control compared to environmental factors such as exercise.

## Discussion

We generated a longitudinal healthcare resource, by comprehensively collecting anthropometric information, 3-month-spanning lifelog, including dietary intake and physical activities, using smartphones and wearable devices, and genetic profiles from 259 participants with the aim of building a personalized genome-based body fat management platform. We demonstrated the potential smartphone- and wearable device-based body fat reduction programs in improving health status, as reflected in the improvements of anthropometric and blood chemistry indicators.

By integrating lifelog and genomic data, we could construct the personal quantified health resources. Since lifestyle and genetic predisposition are critical factors for several disorders, including obesity^4,5,20,32–35^, sufficient variation in lifestyle changes should be induced to estimate the effects of SNPs. In the present study, we varied the lifestyle by dividing the participants into four different groups. Earlier studies intervened with the nutrient intake, physical activity, or sedentary lifestyle of the participants^35,36^. However, such interventions were challenged by the difficulty in continuous monitoring of lifestyle records. Some recent studies attempted either constant monitoring of participant glucose levels or tracking their daily activity using medical devices^36,37^. With wearable devices and regular check-ups/counsel from nutritionists, we were able to collect reliable lifelog resources that concerned diet and physical activity.

The intensity of physical activity in the Han population was shown to modify the effect of genetic risk score (GRS) consisting of 28 BMI-related SNPs, suggesting that the effect of GRS is larger in the group of individuals with low physical activity, compared to those with high physical activity^33^. Therefore, a deeper understanding of the interaction between genetic factors and lifestyle changes may be considered to devise personalized strategies for controlling obesity using genetic profiles. A 3.6-year follow-up study observed patterns similar to ours. This study proposed GRS with six polymorphisms in the FTO gene and their effect on dietary intake patterns, BMI changes, and waist size. Increased consumption of western-style foods, such as fast foods and soft drinks, was associated with a prominent BMI increase in subjects with higher GRS, compared to subjects with lower GRS (P-value for interaction = 0.01). The interaction between the GRS and western-style food consumption was significant for waist size changes (P-value for interaction < 0.05)^38^.

Therefore, similar to the models used for other complex diseases^39–41^ and considering the cumulative effects of common genetic variants, we used GRS here. The personalized genetic test for the GRS-based prediction of either disease risk or phenotypic changes has been popularized by several corporations, such as 23andMe, DNA fit, and Pathway Genomics. Since the personalized risk of obesity could not be explained by significant monogenic mutations^39,42^, GRS, which varied based on personal genetic predispositions, has been used to predict the individual BFM. We used GRS to estimate the effect of the personal genomic features on BFM, corresponding to changes in lifestyles, using GWAS data collected from three groups: GRS-C, -F, and -E.

Although our study suggests a promising novel strategy for personalized genome-guided lifestyle improvement, there are still several points that could be improved. First, the predisposition of the effectiveness of lifestyle change on body fat mass based on their genetic profile was tested in this study. However, the number of participants in the study was small, so it was not sufficient to fully explain the polygenic effect on body fat mass influenced by lifestyle. Second, the lifelog data that we collected contain some missing values. A gradual decrease in compliance on record lifelog data was observed toward the end of the observation period. We asked the participants to provide maximal information on meals during the study to estimate the lifestyle of the baseline and observation periods. The mean number of uploaded days of participants was 55.1 days. Third, unlike whole genome sequencing (WGS), DNA microarray could not completely represent the genetic information of an individual. Therefore, some rare genetic mutations that cause severe obesity or metabolic problems might have been missed in the GRS model. Investigating WGS would accurately represent a personalized healthcare platform. This would provide a personal genetic predisposition and precise genetic mutation causing disease. The result of genetic effects of lifestyle modification on BFM in the study is restricted to the Asian population. Genetic susceptibility to obesity is related to the ethnic background since the relevant BMI per allele in Europeans is greater than in Asians (0.17 kg/m^2^ and 0.11 kg/m^2^ per additional risk allele)^33,43^. As a few studies have considered racial differences, multi-ethnic large-scale studies, using the same SNP set, should be conducted to estimate the accurate interactions between these parameters. Recruiting participants interested in body fat mass change study may induce selection bias as collecting healthier than the average population. However, the mean BMI of participants is 24.81 (s.d. 3.17) which is similar to BMI of 24.57 (s.d. 3.15) from The Korean Genome and Epidemiology Study (KoGES) Consortium^21^. So the bias is less likely to affect the result. Along with this, the recommendation of volunteers who had BMI of 25 to participate in exercise modification group rather than nutrient modification group was suggested because the reduction of nutrients intake may result in worsening their health condition, however, it may also introduce selection bias. Also, there could be an additional bias because the type of intervention can be chosen by the subjects. Although we adjusted the participants’ demographic information as a covariate in the analysis, there may still be a confounding effect that was not considered. One of the potential confounding factors could be seasonal variation. Since the seasons change during the 3 months in which the subjects participate, body fat mass may change without special dietary control. In order to minimize this effect, rather than focusing on the change in body fat mass itself, we focused on the difference in the amount of change between the GRS groups. The enrollment date for all participants was within one week to ensure that all participants passed the same seasonal change. We note that if other researchers want to use this data for other purposes in the future, it should be used in consideration of the seasonal change between 3 months. Finally, BFM, body fat percentage, and BMI varied based on their baseline lifestyle. Anthropometric measurements before participating in the study showed healthier values for those who actively participated in the study, and it may suspect a regular lifestyle of the high compliance group. However, despite the compliance of participants in the study, both the high compliance and low compliance groups improved their health indicators through life modification (Supplementary Fig. S1). Besides, small sample size, selection bias induced from recruiting and selecting lifestyle modification groups, and residual confounding may limit the external validity of the result. Ultimately this represents the need for designing the study with sufficient sample size and controls of selection and confounding bias to highlight the interaction between genetic variants and environmental factors affecting obesity.

Taken together, our findings clarify that genetic factors affect the efficiency of the lifestyle modification-induced BFM changes. We investigated whether genetic factors, environmental factors, and the interaction of both play a role in determining the phenotypes, not only in obesity or BFM control but also in many other traits or diseases. Future studies will seek to explore the application of wearable devices tracking the lifestyle and individual genome to health care and expand the studies in non-European populations.

## Supplementary Information

Supplementary Information.
