# Supplementary material for: Interaction of genetic and environmental factors for body fat mass control: observational study for lifestyle modification and genotyping
Source: Sci Rep. 2021 Jun 23;11:13180. doi: 10.1038/s41598-021-92229-5 (PMC8222320; doi:10.1038/s41598-021-92229-5)
Supplement: Supplementary file 1 — Supplementary Information. [file 41598_2021_92229_MOESM1_ESM.docx]

Interaction of genetic and environmental factors for body fat mass control: observational study for lifestyle modification and genotyping

Joon Ho Kang^1,2, ¶^, Heewon Kim^2, ¶^, Jinki Kim^4, ¶^, Jong-Hwa Seo^4^, Soyeon Cha^2^, Hyunjung Oh^2^, Kyunga Kim^6^, Seong-Jin Park^4^, Eunbin Kim^8^, Sunga Kong^9^, Jae-Hak Lee^2^, Joon Seol Bae^2^, Hong-Hee Won^5^, Je-Gun Joung^6^, Yoon Jung Yang^*,3^, Jinho Kim^*,2^, and Woong-Yang Park^*,1,2,5,6,7^

^1^Department of Molecular Cell Biology, Sungkyunkwan University School of Medicine, 2066 Seobu-ro, Jangan-gu, Suwon, Gyeonggi-do 16419, South Korea

^2^Samsung Genome Institute, Samsung Medical Center, Ilwon-ro 81, Gangnam-gu, Seoul 06351, South Korea

^3^Department of Foods and Nutrition, College of Natural Sciences, Dongduk Women’s University, 60, Hwarang-ro 13-gil, Seongbuk-gu, Seoul 02748, Korea

^4^AI&SW Center, SAIT, SEC, 130, Samsung-ro, Yeongtong-gu, Suwon-si, Gyeonggi, 16678, South Korea

^5^Samsung Advanced Institute for Health Sciences and Technology, Sungkyunkwan University of Medicine, Seoul 06351, South Korea

^6^Samsung Medical Center, Gangnam-gu, Seoul 06351, South Korea

^7^Geninus Inc. Seoul, 5th floor, Jeongeu-ro 70, Songpa-gu, Seoul 05836, South Korea

^8^Department of Clinical Nutrition, School of Public Health, Dongduk Women’s University, Seoul 02748, Korea

^9^Department of Clinical Research Design and Evaluation, SAIHST, Sungkyunkwan University, Seoul, South Korea

**¶**These authors contributed equally to this work.

* Correspondence should be addressed to Yoon jung Yang ([yjyang@dongduk.ac.kr](mailto:yjyang@dongduk.ac.kr)), Jinho Kim ([jinho.jk.kim@samsung.com](mailto:jinho.jk.kim@samsung.com)), or Woong-Yang Park ([woongyang.park@samsung.com](mailto:woongyang.park@samsung.com)).


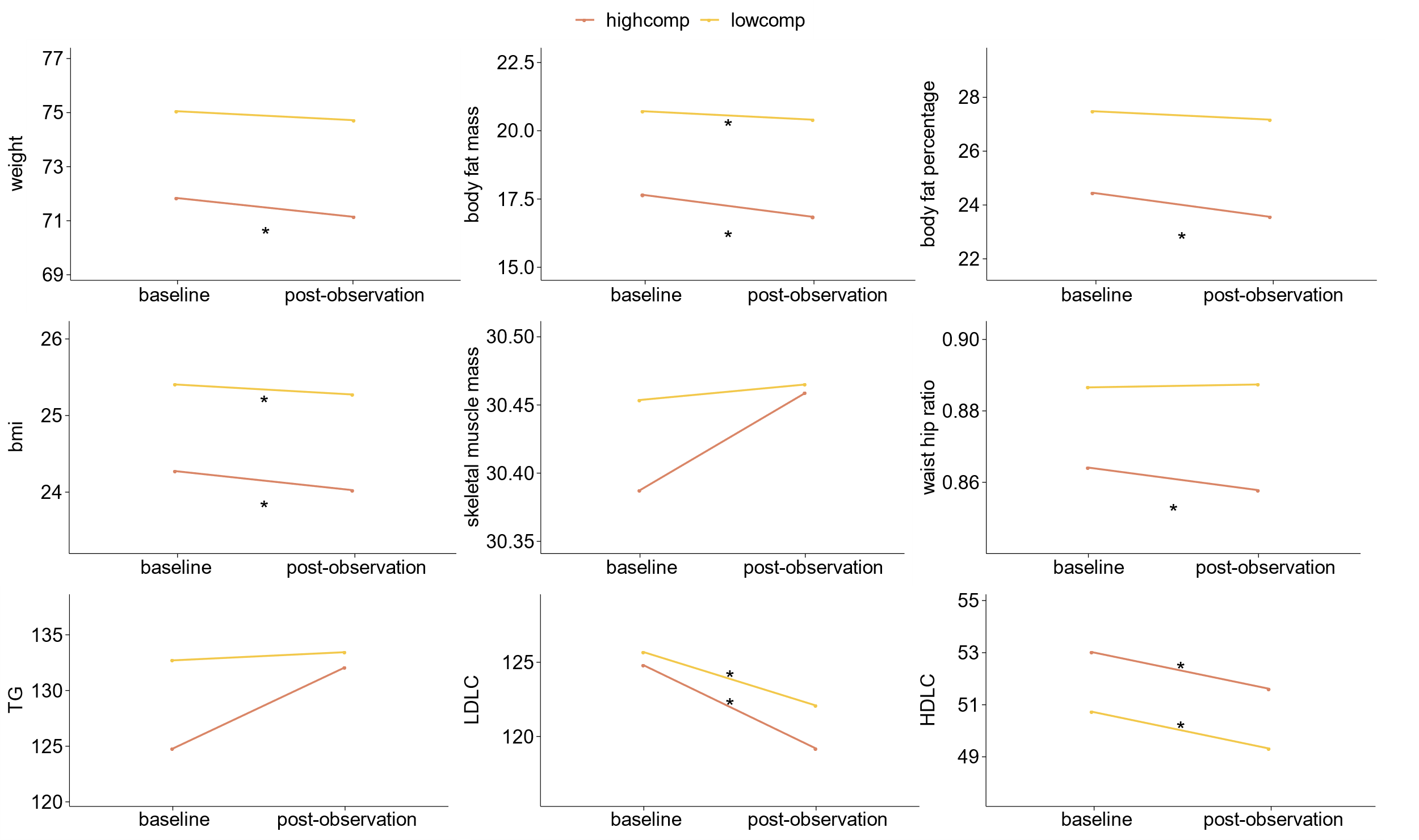


**Supplementary Figure 1. Differences in the anthropometric measurements and serologic markers between the baseline and post-observation period according to compliance** The health indicators improved further through lifestyle modification. Each indicator showed healthier values in the high compliance group both during the baseline and post-observation periods.

|  |  | Adjustment Groups | | | | | | | | | | |
| --- | --- | --- | --- | --- | --- | --- | --- | --- | --- | --- | --- | --- |
|  |  | Low carbohydrate  (n = 35) | |  | Low fat (n = 34) | |  | Moderate exercise (n = 99) | |  | Intense exercise (n = 83) | |
|  |  | Mean | s.d. |  | Mean | s.d. |  | Mean | s.d. |  | Mean | s.d. |
| Age |  | 39.66 | 7.50 |  | 37.24 | 6.32 |  | 36.92 | 7.10 |  | 36.58 | 7.96 |
| Body fat mass (kg) |  | 22.81 | 4.99 |  | 24.47 | 4.08 |  | 17.55 | 6.28 |  | 16.77 | 4.53 |
| Body fat percentage (%) |  | 28.23 | 6.20 |  | 29.23 | 3.79 |  | 24.95 | 6.53 |  | 24.51 | 5.53 |
| Body mass index (kg/m^2^) |  | 27.27 | 1.75 |  | 27.77 | 1.80 |  | 23.87 | 3.18 |  | 23.39 | 2.46 |
| Skeletal muscle mass (kg) |  | 32.93 | 4.94 |  | 33.41 | 3.42 |  | 29.37 | 6.61 |  | 28.91 | 5.66 |
|  |  | Baseline | Observation |  | Baseline | Observation |  | Baseline | Observation |  | Baseline | Observation |
| Carbohydrate intake (g/day) |  | 200.41 | 176.40 |  | 210.64 | 198.27 |  | 200.50 | 200.50 |  | 204.96 | 193.20 |
| Fat intake (g/day) |  | 57.68 | 53.95 |  | 56.91 | 40.04 |  | 54.23 | 52.12 |  | 53.06 | 50.12 |
| Total calorie (cal/day) |  | 1699.00 | 1545.00 |  | 1717.00 | 1459.30 |  | 1658.00 | 1631.70 |  | 1694.60 | 1602.10 |
| Exercise amount (cal/day) |  | 252.20 | 213.60 |  | 202.80 | 194.18 |  | 177.79 | 200.54 |  | 228.92 | 260.13 |

**Supplementary Table 1. Demographic information and lifestyle of the participants during the baseline and observation periods** Anthropometric measurements of each adjustment group are measured. The nutrient intake during the observation period got reduced compared to the baseline period. The exercise amount of the exercise modification group increased throughout the observation period.

| SNP set for GRS consturction | | |
| --- | --- | --- |
| Category | SNP | Gene |
| Carbohydrate | rs206936 | RPS10-NUDT3 |
| Carbohydrate | rs7578465 | ALK |
| Carbohydrate | rs7920888 | intergenic |
| Carbohydrate | rs13041126 | intergenic |
| Carbohydrate | rs2198776 | FAM19A2 |
| Carbohydrate | rs2391518 | intergenic |
| Carbohydrate | rs7143963 | TRAF3 |
| Carbohydrate | rs2972167 | PPARG |
| Carbohydrate | rs17036333 | PPARG |
| Carbohydrate | rs253664 | MRAS |
| Carbohydrate | rs492400 | USP37 |
| Carbohydrate | rs1557765 | intergenic |
| Carbohydrate | rs148679664 | AGT |
| Carbohydrate | rs10742752 | intergenic |
| Carbohydrate | rs11997175 | intergenic |
| Carbohydrate | rs9856151 | CPNE4 |
| Carbohydrate | rs4061073 | SSBP3 |
| Carbohydrate | rs11603334 | ARAP1 |
| Carbohydrate | rs13077495 | PPARG |
| Carbohydrate | rs17793951 | PPARG |
| Carbohydrate | rs189428681 | PPARG |
| Carbohydrate | rs191018871 | PPARG |
| Carbohydrate | rs6782178 | PPARG |
| Carbohydrate | rs6810295 | OSBPL10 |
| Carbohydrate | rs10132280 | intergenic |
| Carbohydrate | rs2920502 | PPARG |
| Carbohydrate | rs5050 | AGT |
| Carbohydrate | rs10760279 | intergenic |
| Carbohydrate | rs2972164 | PPARG |
| Carbohydrate | rs12885454 | intergenic |
| Carbohydrate | rs12646911 | GLRA3 |
| Carbohydrate | rs11677911 | intergenic |
| Carbohydrate | rs12518350 | intergenic |
| Carbohydrate | rs652722 | DKFZp686K1684 |
| Carbohydrate | rs12401322 | APOA2 |
| Carbohydrate | rs11142387 | intergenic |
| Carbohydrate | rs2237892 | KCNQ1 |
| Fat | rs2237892 | KCNQ1 |
| Fat | rs11122577 | AGT |
| Fat | rs7551318 | intergenic |
| Fat | rs4243830 | PLEKHG5 |
| Fat | rs4144743 | intergenic |
| Fat | rs2478545 | AGT |
| Fat | rs10889850 | intergenic |
| Fat | rs1413020 | intergenic |
| Fat | rs2972165 | PPARG |
| Fat | rs2389438 | intergenic |
| Fat | rs2938398 | PPARG |
| Fat | rs11142387 | intergenic |
| Fat | rs9473924 | intergenic |
| Fat | rs1546924 | FAM212B |
| Fat | rs10136789 | KCNH5 |
| Fat | rs2972164 | PPARG |
| Fat | rs2365389 | FHIT |
| Fat | rs10769908 | STK33 |
| Fat | rs11603334 | ARAP1 |
| Exercise | rs7164727 | intergenic |
| Exercise | rs11583200 | ELAVL4 |
| Exercise | rs1205 | CRP |
| Exercise | rs2845885 | MACROD1 |
| Exercise | rs3093059 | CRP |
| Exercise | rs13076933 | PPARG |
| Exercise | rs1555543 | intergenic |
| Exercise | rs10136789 | KCNH5 |
| Exercise | rs2124499 | ADCY5 |
| Exercise | rs657452 | AGBL4 |
| Exercise | rs7481311 | BDNF-AS1 |
| Exercise | rs11568020 | AGT |
| Exercise | rs4432245 | EIF2AK4 |
| Exercise | rs10929925 | intergenic |
| Exercise | rs4762 | AGT |
| Exercise | rs2770102 | LINC00340 |
| Exercise | rs2228213 | HIVEP1 |
| Exercise | rs2921186 | PPARG |
| Exercise | rs7611238 | ACAP2 |
| Exercise | rs12143241 | APOA2 |
| Exercise | rs5085 | APOA2 |
| Exercise | rs10913469 | SEC16B |
| Exercise | rs6465468 | ASB4 |
| Exercise | rs206936 | RPS10-NUDT3 |
| Exercise | rs11787111 | intergenic |

**Supplementary Table 2. SNP for constructing GRS-C, F and E** SNP sets used for GRS construction.
